# Abnormal immunity of non-survivors with COVID-19: predictors for mortality

**DOI:** 10.1186/s40249-020-00723-1

**Published:** 2020-08-03

**Authors:** Yang Zhao, Han-Xiang Nie, Ke Hu, Xiao-Jun Wu, Yun-Ting Zhang, Meng-Mei Wang, Tao Wang, Zhi-Shui Zheng, Xiao-Chen Li, Shao-Lin Zeng

**Affiliations:** Department of Respiratory Medicine, Renmin Hospital of Wuhan University, 238 Jiefang Road, Wuchang District, Wuhan, 430060 China

**Keywords:** COVID-19, Cellular immunity, Humoral immunity, Mortality

## Abstract

**Background:**

The number of coronavirus disease 2019 (COVID-19) cases has rapidly increased all over the world. Specific information about immunity in non-survivors with COVID-19 is scarce. This study aimed to analyse the clinical characteristics and abnormal immunity of the confirmed COVID-19 non-survivors.

**Methods:**

In this single-centered, retrospective, observational study, we enrolled 125 patients with COVID-19 who were died between January 13 and March 4, 2020 in Renmin Hospital of Wuhan University. A total of 414 randomly recruited patients with confirmed COVID-19 who were discharged from the same hospital during the same period served as control. The demographic, clinical characteristics and laboratory findings at admission, and treatment used in these patients were collected. The immunity-related risk factors associated with in-hospital death were tested by logistic regression models and Receiver Operating Characteristic (ROC) curve.

**Results:**

Non-survivors (70 years, IQR: 61.5–80) were significantly older than survivors (54 years, IQR: 37–65) (*P* <  0.001). 56.8% of non-survivors was male. Nearly half of the patients (44.9%) had chronic medical illness. In non-survivors, hypertension (49.6%) was the most common comorbidity, followed by diabetes (20.0%) and coronary heart disease (16.0%). The common signs and symptoms at admission of non-survivors were fever (88%), followed by cough (64.8%), dyspnea (62.4%), fatigue (62.4%) and chest tightness (58.4%). Compared with survivors, non-survivors had higher white blood cell (WBC) count (7.85 vs 5.07 × 10^9^/L), more elevated neutrophil count (6.41 vs 3.08 × 10^9^/L), smaller lymphocyte count (0.69 vs 1.20 × 10^9^/L) and lower platelet count (172 vs 211 × 10^9^/L), raised concentrations of procalcitonin (0.21 vs 0.06 ng/mL) and CRP (70.5 vs 7.2 mg/L) (*P* < 0.001). This was accompanied with significantly decreased levels of CD3^+^ T cells (277 vs 814 cells/μl), CD4^+^ T cells (172 vs 473 cells/μl), CD8^+^ T cells (84 vs 262.5 cells/μl, *P* < 0.001), CD19^+^ T cells (88 vs 141 cells/μl) and CD16^+^ 56^+^ T cells (79 vs 128.5 cells/μl) (*P* < 0.001). The concentrations of immunoglobulins (Ig) G (13.30 vs 11.95 g/L), IgA (2.54 vs 2.21 g/L), and IgE (71.30 vs 42.25 IU/ml) were increased, whereas the levels of complement proteins (C)3 (0.89 vs 0.99 g/L) and C4 (0.22 vs 0.24 g/L) were decreased in non-survivors when compared with survivors (all *P* < 0.05). The non-survivors presented lower levels of oximetry saturation (90 vs 97%) at rest and lactate (2.40 vs 1.90 mmol/L) (*P* < 0.001). Old age, comorbidity of malignant tumor, neutrophilia, lymphocytopenia, low CD4^+^ T cells, decreased C3, and low oximetry saturation were the risk factors of death in patients with confirmed COVID-19. The frequency of CD4^+^ T cells positively correlated with the numbers of lymphocytes (*r* = 0.787) and the level of oximetry saturation (*r* = 0.295), Whereas CD4^+^ T cells were negatively correlated with age (*r* =-0.323) and the numbers of neutrophils (*r* = − 0.244) (all *P* < 0.001).

**Conclusions:**

Abnormal cellular immunity and humoral immunity were key features of non-survivors with COVID-19. Neutrophilia, lymphocytopenia, low CD4^+^ T cells, and decreased C3 were immunity-related risk factors predicting mortality of patients with COVID-19.

## Introduction

Coronavirus disease 2019 (COVID-19) has declared as a pandemic on 12 March, 2020. The pathogen has been identified as a novel enveloped RNA beta-coronavirus that is currently known as severe acute respiratory syndrome coronavirus 2 (SARS-CoV-2, also called 2019-nCoV) [1]. Like severe acute respiratory syndrome (SARS) coronavirus and Middle East respiratory syndrome (MERS) coronavirus, SARS-CoV-2 is a highly pathogenic coronavirus to human. The virus has high transmission capability as well as high morbidity and mortality [2–4]. By April 22, 2020, the acute respiratory disease has affected more than 200 countries around the world. Globally, 2 553 853 cases have been confirmed, with 176 323 deaths have died from this viral infection [5]. However, little is still known about the factors that lead to death by this coronavirus.

Recent reports show that the severely ill patients with confirmed COVID-19 may develop dyspnea and hypoxemia within 1 week after the onset of disease, which may quickly progress to acute respiratory distress syndrome (ARDS) or end-organ failure [6, 7]. The general epidemiological features and clinical characteristics of patients with COVID-19 have been previously reported [2, 4, 8]. However, these studies were based on relatively small sample sizes, specific characterizations of the abnormal immunity of non-survivors with COVID-19 have not yet been well described. In this study, we aimed to explore the clinical characteristics and immunity features in patients with COVID-19 from a hospital in Wuhan, China and evaluate the immunity-related risk factors associated with patient death.

## Method

### Patients’ involvement and data collection

In this retrospective, single center study, a total of 125 patients who died from confirmed COVID-19 between January 13 and March 4, 2020 in Renmin Hospital of Wuhan University, the designated hospital for treating severe patients with COVID-19 in Wuhan, China. A total of 414 randomly recruited patients with confirmed COVID-19 who were discharged from the same hospital during the same period served as control. All patients were confirmed of COVID-19 according to the World Health Organization interim guidance [9] and Diagnosis and Treatment Guideline for Novel Coronavirus Pneumonia (Trial Version 7.0) [10], and were positive for at least two nucleic acid tests for SARS-CoV-2. The confirmed COVID-19 patients were classified as severe or non-severe cases based on the American Thoracic Society guidelines for community-acquired pneumonia [11]. The patients who received systemic corticosteroid treatment before admission were excluded. The criteria for patient discharge included absence of fever for at least 3 days, substantial improvement in both lungs in chest computed tomography (CT), clinical remission of respiratory symptoms, and negative results for two throat-swab samples obtained at least 24 h apart for SARS-CoV-2 RNA testing.

This study was approved by the Research Ethics Commission of Renmin Hospital of Wuhan University (No.WDRY2020-K143), and written informed consent was waived by the Research Ethics Commission.

Demographic and clinical characteristics (including comorbidities, signs and symptoms), laboratory findings, chest CT scan results and treatment were extracted from electronic medical records system of Renmin Hospital of Wuhan University and analysed by three independent researchers. The access was granted by the director of the hospital. The date of disease onset and admission date, as well as the severity of COVID-19, were also recorded. None of these cases have been previously reported.

### Laboratory testing

Patient pharyngeal swab specimens were collected for SARS-CoV-2 nucleic acid detection using real-time reverse transcriptase-polymerase chain reaction (RT-PCR) assay with a standard procedure recommended by Chinese Center for Disease Control and Prevention (China CDC). The viral nucleic acid testing for all patients was performed by the clinical laboratory from Renmin Hospital of Wuhan University, a certified laboratory for SARS-CoV-2 testing. Other laboratory tests at admission, included white blood cell (WBC), neutrophil, lymphocyte, platelet, procalcitonin (PCT), C-reactive protein (CRP), CD3^+^ T cells, CD4^+^ T cells, CD8^+^ T cells, CD19^+^ T cells, CD16^+^ 56^+^ T cells, immunoglobulins (Ig) G, IgM, IgA, IgE, complement proteins (C)3, C4, oximetry saturation, and lactate, were performed in the same hospital and results were retrieved from electronic medical records. Some cytokines such as interleukin (IL)-6, interferon-γ, IL-10 and other cytokines, were not performed in all patients.

### Statistical analysis

All statistical analyses were performed using SPSS 24.0 software (SPSS Inc., Chicago, USA). Categorical variables were expressed with frequencies and percentages. Continuous variables were presented as median (interquartile range [IQR]). Means for continuous variables were compared with independent-samples t tests when the data were normally distributed; if not, the Mann-Whitney U test was used. Proportions for categorical variables were compared with the chi-square and Fisher’s exact test. Univariable logistic regression between basic disease or different parameter and demise was performed. Moreover, the main risks related with demise were examined using multivariable logistic regression models adjusted for potential confounders. Receiver Operating Characteristic (ROC) curve model was also used to predict death efficacy. Spearman’s correlation test was used for calculation of correlation between different factors. Statistical significance was determined at *P* < 0.05.

## Results

### Demographic and clinical characteristics

A total of 539 patients with confirmed COVID-19 (125 non-survivors and 414 survivors) with comprehensive medical records were included in this study. All patients had pneumonia with abnormal findings on their chest CT scan. The median age of all patients was 58.0 years (IQR: 43.0–69.0), ranging from 16 years to 97 years.

Non-survivors (70 years, IQR: 61.5–80) were significantly older than survivors (54 years, IQR: 37–65) (*P* <  0.001). Of all COVID-19 patients, the majority was female (52.7%). More than half of non-survivors were male (56.8%). Nearly half of the COVID-19 patients (44.9%) had chronic medical illness, with hypertension being the most common comorbidity, followed by diabetes, coronary heart disease and malignant tumour. In non-survivors, hypertension (49.6%) was the most common comorbidity, followed by diabetes (20.0%) and coronary heart disease (16.0%). The median interval from illness onset to death was 7.0 days (IQR: 4.0–11.0), which is markedly lower than the median time from illness onset to discharge 15 days (IQR: 9.0–21.0). The common signs and symptoms at admission of non-survivors were fever (88%), followed by cough (64.8%), dyspnea (62.4%), fatigue (62.4%) and chest tightness (58.4%).

As for clinical classification, the patients were divided into severe group and non-severe groups. Severe patients (98.4%) were more likely to die compared with non-severe patients and nearly all of non-survivors were classified as severe cases at admission. Almost all the non-survivors received antibiotic treatment (94.4%). Both groups of patients were given antiviral therapy or Chinese Medicine, such as Lianhua Qingwen, oseltamivir, arbidol, and lopinavir/ritonavir. Intravenous human immunoglobulin and corticosteroid were used more frequently in non-survivors than survivors (Table 1).

**Table 1:** Demographics, clinical characteristics and treatment of patients with COVID-19

	Total (539)	Non-survivor (125)	Survivor (414)	*P* value
Age (years)	58 (43–69)	70 (61.5–80)	54 (37–65)	< 0.0001
Gender				0.015
Male	255 (47.3%)	71 (56.8%)	184 (44.4%)	
Female	284 (52.7%)	54 (43.2%)	230 (55.6%)	
Chronic medical illness	242 (44.9%)	88 (70.4%)	154 (37.2%)	< 0.0001
Chronic obstructive lung disease	22 (4.1%)	12 (9.6%)	10 (2.4%)	< 0.0001
Diabetes	58 (10.9%)	25 (20.0%)	34 (8.2%)	< 0.0001
Hypertention	140 (26.0%)	62 (49.6%)	78 (18.8%)	< 0.0001
Coronary heart disease	37 (6.9%)	20 (16.0%)	17 (4.1%)	< 0.0001
Cerebrovascular diseases	19 (3.5%)	15 (12.0%)	4 (1.0%)	< 0.0001
Hepatitis	11 (2.0%)	3 (2.4%)	8 (1.9%)	0.746
Malignant tumour	23 (4.3%)	11 (8.8%)	12 (2.9%)	0.0042
Chronic kidney disease	8 (1.5%)	6 (4.8%)	2 (0.5%)	0.0005
Immunodeficiency disease	2 (0.4%)	0 (0.0%)	2 (0.5%)	0.436
Time from illness onset to death or discharge, days	13 (7–20)	7 (4–11)	15 (9–21)	< 0.0001
Signs and symptoms at admission				
Fever	420 (77.9%)	110 (88%)	310 (74.9)	0.001
Rhinorrhoea	6 (1.1%)	2 (1.6%)	4 (1.0%)	0.945
Cough	332 (61.6%)	81 (64.8%)	251 (60.6%)	0.402
Expectoration	161 (29.9%)	48 (38.4%)	113 (27.3%)	0.017
Chest tightness	187 (34.7%)	73 (58.4%)	114 (27.5%)	< 0.0001
Dyspnea	187 (34.7%)	78 (62.4%)	98 (23.7%)	< 0.0001
Fatigue	261 (48.4%)	78 (62.4%)	183 (44.2%)	< 0.0001
Nausea	9 (1.7%)	5 (4.0%)	4 (1.0%)	0.02
Diarrhea	58 (10.8%)	8 (6.4%)	50 (12.1%)	0.073
Clinical Classification				< 0.0001
Non-severe	133 (24.7%)	2 (1.6%)	131 (31.6%)	
Severe	406 (75.3%)	123 (98.4%)	283 (68.4%)	
Treatment				
Antibiotic drug	374 (69.4%)	118 (94.4%)	256 (61.8%)	< 0.0001
Antiviral therapy or Chinese Medicine	225 (41.7%)	58 (46.4%)	167 (40.3%)	0.228
Lianhua Qingwen	301 (55.8%)	40 (32.0%)	261 (63.0%)	< 0.0001
Oseltamivir	157 (29.1%)	48 (38.4%)	109 (26.3%)	0.0092
Arbidol	370 (68.6%)	65 (52.0%)	305 (73.7%)	< 0.0001
Lopinavir/ritonavir	32 (5.9%)	18 (14.4%)	14 (3.4%)	< 0.0001
Human immunoglobulin	250 (38.4%)	91 (72.8%)	159 (38.4%)	< 0.0001
Alpha-interferon	170 (31.5%)	34 (27.2%)	136 (32.9%)	0.233
Thymosin	59 (10.9%)	20 (16.0%)	39 (9.4%)	0.039
Glucocorticoids	249 (46.2%)	89 (71.2%)	160 (38.6%)	< 0.0001

Data are median (IQR) and *n* (%). *P* values were calculated by Mann-Whitney U test, *χ*^2^ test, or Fisher’s exact test, as appropriate. Statistical significance was determined at *P*<0.05

*COVID-19* Coronavirus disease 2019

### Blood routine examination, cellular immunity, humoral immunity, and other laboratory findings in non-survivors with COVID-19

Although WBC and platelet count of both non-survivors and survivors were in normal range, the non-survivors had higher WBC count (7.85 × 10^9^/L vs 5.07 × 10^9^/L), more elevated neutrophil count (6.41 × 10^9^/L vs 3.08 × 10^9^/L), smaller lymphocyte count (0.69 × 10^9^/L vs 1.20 × 10^9^/L) and lower platelets count (172 × 10^9^/L vs 211 × 10^9^/L) than survivors (*P* < 0.001).

Increased levels of infection-related biomarkers, including PCT (0.21 vs 0.06 ng/ml) and CRP (70.5 vs 7.2 mg/L) were found in non-survivors when compared with those of survivors (*P* < 0.001).

The levels of CD3^+^ T cells (277 vs 814 cells/μl), CD4^+^ T cells (172 vs 473 cells/μl), CD8^+^ T cells (84 vs 262.5 cells/μl, *P* < 0.001), CD19^+^ T cells (88 vs 141 cells/μl) and CD16^+^ 56^+^ T cells (79 vs 128.5 cells/μl) were significantly decreased in non-survivors when compared with survivors (*P* < 0.001).

The serum concentrations of IgG (13.30 vs 11.95 g/L, *P* < 0.001), IgA (2.54 vs 2.21 g/L, *P* = 0.012), and IgE (71.30 vs 42.25 IU/ml, *P* < 0.001) were increased, whereas the serum levels of C3 (0.89 vs 0.99 g/L, *P* < 0.001) and C4 (0.22 vs 0.24 g/L, *P* = 0.001) were decreased in non-survivors when compared with survivors. The non-survivors presented lower levels of resting oximetry saturation (90 vs 97%, *P* < 0.001) and lactate (2.40 vs 1.90 mmol/L, *P* < 0.001) (Table 2). IL-6 concentrations test was just performed in only 135 patients, and the available data illustrated that the levels of IL-6 in non-survivors were significantly higher than survivors (*P* < 0.001) (Table 3).

**Table 2:** Laboratory findings of patients with COVID-19

	Normal Range	Total (539)	No-survivor (125)	Survivor (414)	*P* value
WBC (× 10^9^/L)	3.5–9.5	5.41 (4.1–7.59)	7.85 (4.74–12.01)	5.07 (3.96–6.76)	< 0.001
Neutrophils (× 10^9^/L)	1.8–6.3	3.54 (2.42–5.72)	6.41 (3.77–10.97)	3.08 (2.29–4.63)	< 0.001
Lymphocytes (× 10^9^/L)	1.1–3.2	1.04 (0.76–1.5)	0.69 (0.42–0.93)	1.20 (0.88–1.63)	< 0.001
Haemoglobin (g/L)	115–150	126 (115–138)	127 (113.5–139.5)	126 (116–137)	0.761
Platelets (× 10^9^/L)	125–350	205 (151–261)	172 (118–235)	211 (163–271)	< 0.001
CRP (mg/L)	0–10	16.9 (5–61.3)	70.5 (39.95–133.5)	7.2 (5–37.95)	< 0.001
PCT (ng/mL)	< 0.1	0.07 (0.04–0.25)	0.21 (0.11–0.95)	0.06 (0.03–0.12)	< 0.001
CD3^+^ T cells (cells/μl)	723–2737	667 (356–1010)	277 (163.5–430)	814 (516–1088)	< 0.001
CD4^+^ T cells (cells/μl)	404–1612	392 (200–586)	172 (99.5–267.5)	473 (291–657.75)	< 0.001
CD8^+^ T cells (cells/μl)	220–1129	221 (104–366)	84 (39.5–155.5)	262.5 (163–405.25)	< 0.001
CD19^+^T cells (cells/μl)	80–616	132 (82–202)	88 (52–151)	141 (96–219)	< 0.001
CD16^+^ 56^+^ T cells (cells/μl)	84–724	114 (70–190)	79 (39–144)	128.5 (79–210)	< 0.001
IgG (g/L)	7.0–16.0	12.2 (10.3–14.6)	13.30 (10.75–16.95)	11.95 (10.18–13.93)	< 0.001
IgM (g/L)	0.4–2.3	0.95 (0.70–1.3)	0.94 (0.70–1.26)	0.95 (0.69–1.31)	0.658
IgA (g/L)	0.7–4.0	2.26 (1.74–3)	2.54 (1.81–3.46)	2.21 (1.73–2.89)	0.012
IgE (IU/ml)	< 100	52.3 (18.3–133)	71.30 (30.2–214.5)	42.25 (18.3–120)	< 0.001
C3 (g/L)	0.9–1.8	0.97 (0.82–1.1)	0.89 (0.74–1.04)	0.99 (0.84–1.13)	< 0.001
C4 (g/L)	0.1–0.4	0.241 (0.18–0.32)	0.22 (0.159–0.30)	0.24 (0.19–0.32)	0.001
Oximetry saturation (%)	95–100	97 (92–98)	90 (83–94)	97 (95–98)	< 0.001
Lactate (mmol/L)	0.5–1.5	2 (1.5–2.53)	2.40 (1.9–3.65)	1.90 (1.43–2.38)	< 0.001

Data are median (IQR). *WBC* White blood cell, *CRP* C-reactive protein, *PCT* Procalcitonin, *Ig* immunoglobulins, *C* Complement proteins. *P* values were calculated by Mann-Whitney U test, *χ*^2^ test, or Fisher’s exact test, as appropriate. Statistical significance was determined at *P*<0.05

**Table 3:** IL-6 concentrations of patients with COVID-19

	Normal Range	Total (135)	No-survivor (33)	Survivor (102)	*P* value
IL-6 (mg/L)	≤ 20.0	7.22 (5.06–22.5)	60.72 (16.89–146.53)	5.98 (4.63–12.12)	< 0.001

Data are median (IQR)

*P* values were calculated by Mann-Whitney U test. Statistical significance was determined at *P*<0.05

### Associations between immunity-related indexes and death risk of COVID-19 patients

Univariable logistic regression analysis indicated that old age, comorbidity of malignant tumor, neutrophilia, lymphocytopenia, increased CRP levels, lower CD4^+^ T cell count, and decreased C3 levels were associated with death (Table 4).

**Table 4:** Univariable logistic Regression of death risk of patients with COVID-19

	Wald	*P*	*OR*	95% *CI*
Age	27.347	<0.001	0.912	0.881–0.944
Malignant tumour	4.563	0.023	0.176	0.036–0.866
Neutrophils	5.239	0.022	0.881	0.790–0.982
Lymphocytes	7.674	0.003	0.109	0.032–0.186
CRP	4.052	0.044	0.992	0.984–1.000
CD4^+^ T cells	9.147	0.002	1.005	1.002–1.009
C3	8.033	0.005	40.209	3.326–528.035

*OR* Odds ratio, *CI* Confidence interval, *CRP* C-reactive protein, *C* Complement proteins, *COVID-19* Coronavirus disease 2019

Statistical significance was determined at *P*<0.05

Generalized linear model showed that old age, comorbidity of malignant tumor, neutrophilia, lymphocytopenia, lower CD4^+^ T cells, decreased C3 and lower oximetry saturation was positively with the risk of death (Table 5).

**Table 5:** Generalised linear model of patients with COVID-19

	Wald Chi-Sqare	*P*	Standard error
Age	25.182	<0.001	0.0009
Malignant tumour	24.070	<0.001	0.0638
Neutrophils	9.813	0.002	0.0062
Lymphocytes	5.831	0.016	0.0357
CD4^+^ T cells	3.602	0.048	0.00268
C3	6.928	0.008	0.0291
Oximetry saturation	9.555	0.002	0.0017

*C* Complement proteins, *COVID-19* Coronavirus disease 2019

Statistical significance was determined at *P*<0.05

**Fig. 1:**
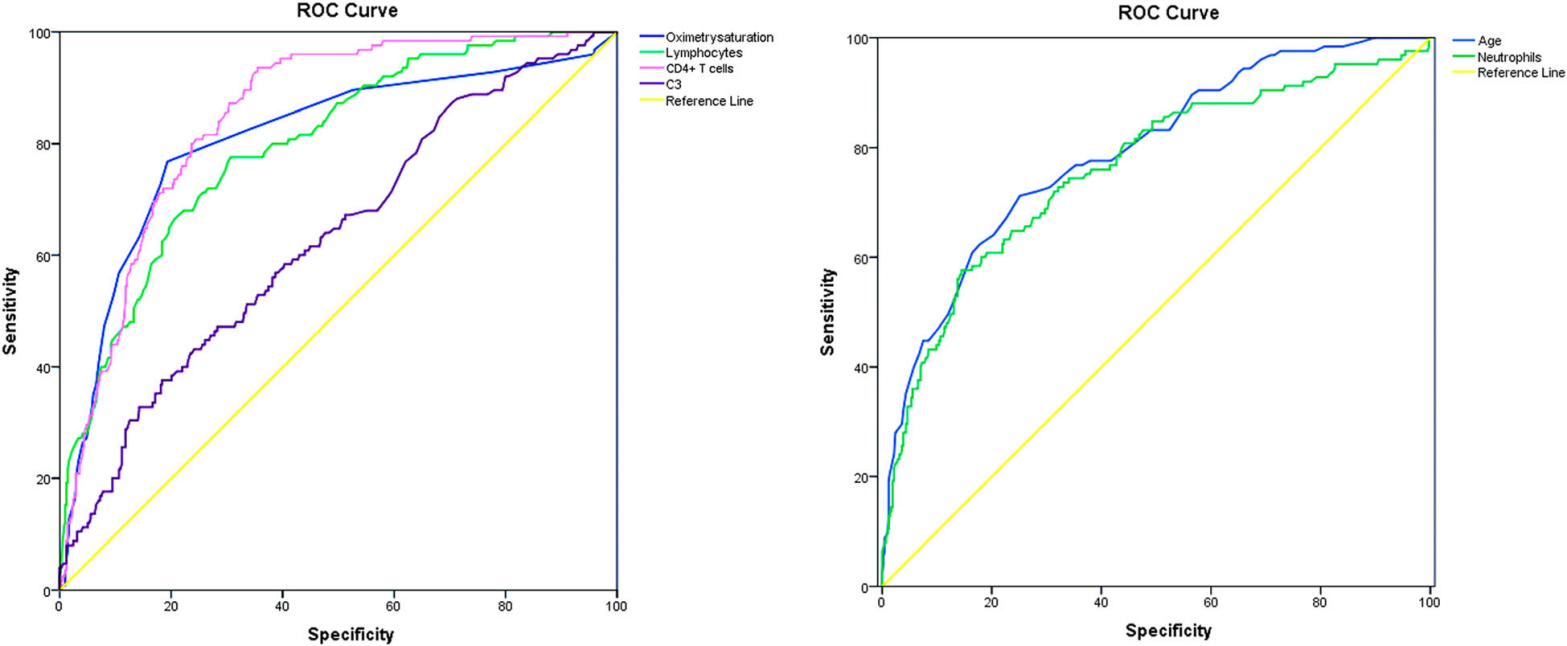
ROC curve model of age, neutrophils, lymphocytes, CD4^+^ T cells, C3 and Oximetry saturation in patients with COVID-19. ROC: Receiver operating characteristic; C: Complement proteins; COVID-19: Coronavirus disease 2019

**Fig. 2:**
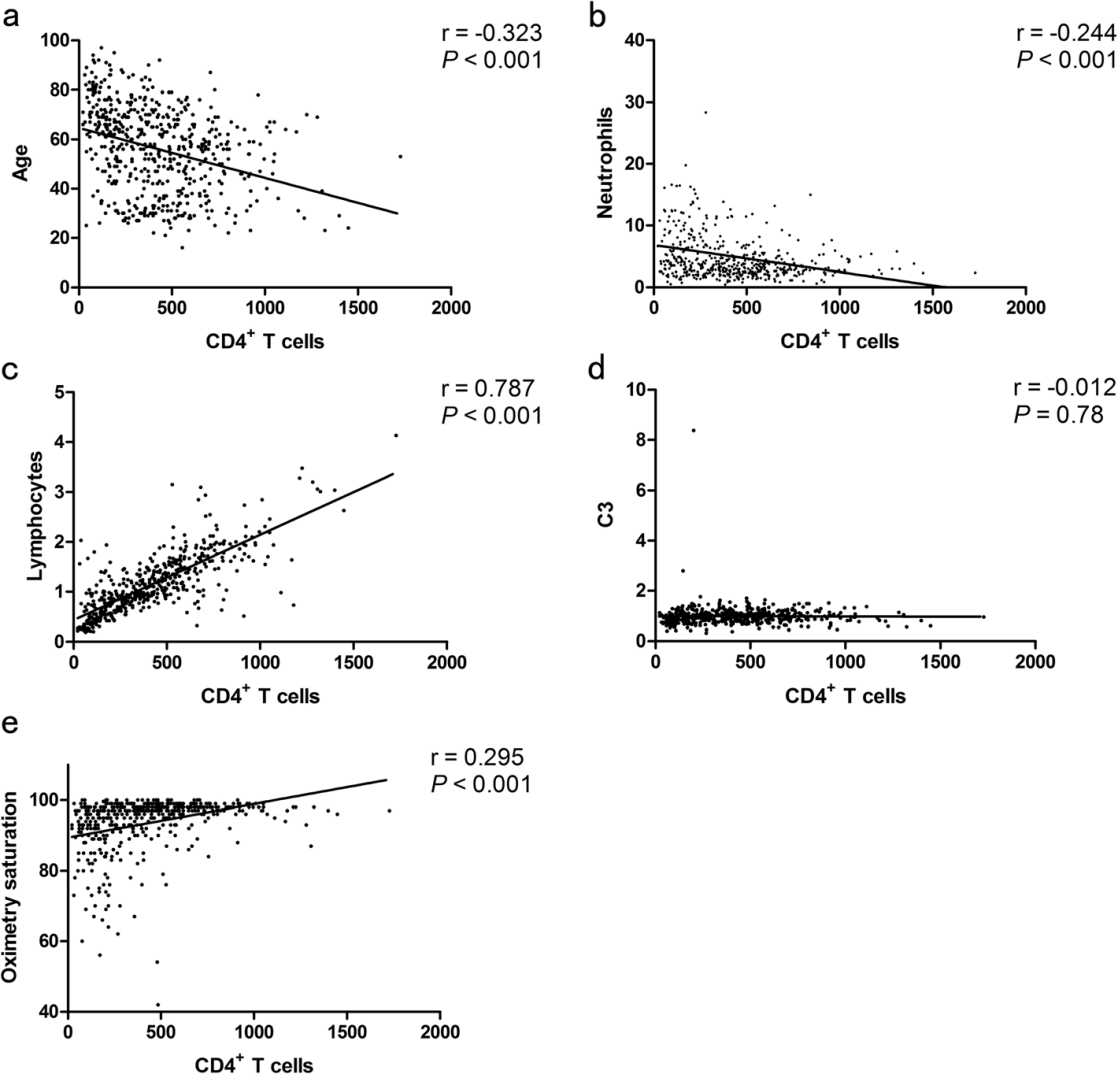
Correlation between CD4^+^ T cells and age. (**a**); CD4^+^ T cells and neutrophils (**b**); CD4^+^ T cells and lymphocytes (**c**); CD4^+^ T cells and C3 (**d**); CD4^+^ T cells and Oximetry saturation (**e**) of COVID-19 patients. Spearman’s test was used to evaluate the correlation. Statistical significance was determined at *P*<0.05. ROC: Receiver operating characteristic; C: Complement proteins; COVID-19: Coronavirus disease 2019

Further, we conducted ROC curve analysis to evaluate the role of these factors in predicting death efficacy. The area under the ROC curve (AUC) was 0.792 (confidence interval [*CI*]: 0.764–0.837) for age, 0.761(95% *CI*: 0.709–0.812) for neutrophils, 0.797 (95% *CI*: 0.753–0.840) for lymphocytes, 0.821 (95% *CI*: 0.782–0.860) for CRP, 0.848 (95% *CI*: 0.814–0.883) for CD4^+^ T cells, 0.630 (95% *CI*: 0.574–0.685) for C3, and 0.808 (95% *CI*: 0.759–0.856) for oximetry saturation. Additionally, the cutoff value was 64.5 for age, 5.835 for neutrophils, 0.945 for lymphocytes, 31.4 for CRP, 380.5 for CD4^+^ T cells, 0.809 for C3, and 94.5 oximetry saturation (Table 6, Fig. 1). The abundance of CD4^+^ T cells was positively correlated with the numbers of lymphocytes (*r* = 0.787, *P* < 0.001) and the level of oximetry saturation (*r* = 0.295, *P* < 0.001), respectively. Whereas it was negatively correlated with age (*r* = -0.323, *P* < 0.001) or the numbers of neutrophils (*r* = − 0.244, *P* < 0.001). However, CD4^+^ T cell abundance showed no significant correlation with C3 levels (*P* = 0.78) (Fig. 2).

**Table 6:** ROC curve model of patients with COVID-19

	Area	Std.error	*P*	95% *CI* lower	95% *CI* upper	Cut off
Age	0.792	0.023	0.000	0.764	0.837	64.5
Neutrophils	0.761	0.026	0.000	0.709	0.812	5.835
Lymphocytes	0.797	0.22	0.000	0.753	0.840	0.945
CRP	0.821	0.020	0.000	0.782	0.860	31.4
CD4^+^ T cells	0.848	0.018	0.000	0.814	0.883	380.5
C3	0.630	0.028	0.000	0.574	0.685	0.809
Oximetry saturation	0.808	0.25	0.000	0.759	0.856	94.5

*ROC* Receiver Operating Characteristic, *CI* Confidence interval, *CRP* C-reactive protein, *C* Complement proteins, *COVID-19* Coronavirus disease 2019

Statistical significance was determined at *P*<0.05

## Discussion

In this study, we showed that the non-survivors with confirmed COVID-19 had higher WBC count, neutrophilia, lymphocytopenia, and lower platelet level when compared with survivors at admission. Abnormal immune responses, represented by dysregulation of numerous cellular and humoral immunity markers were found in non-survivors. Old age, comorbidity of malignant tumor, neutrophilia, lymphocytopenia, low CD4^+^ T cells, decreased C3, and low oximetry saturation were positively correlated with the risk of death in patients with COVID-19.

In this study, we found that non-survivors with confirmed COVID-19 were older, especially above 64.5 years, and more than half were male. Additionally, nearly half of the patients had chronic medical illness with comorbidity of malignant tumor being the risk of death. Previous reports have demonstrated age-dependent defects in T-cell and B-cell function, and excessive production of type 2 cytokines could cause deficiency in control of viral replication and prolonged proinflammatory responses, potentially leading to poor outcome [12]. Furthermore, chronic comorbidities often compromise immune system [13]. In agreement with these previous findings, our data suggested that COVID-19 is more likely to cause death in older men with chronic comorbidities than other patients [14].

In this study, we showed that severely ill patients were more likely to die compared with the non-severe patients even if they were given integrated therapy and that almost all non-survivors were severely ill at admission. Meanwhile, the non-survivors had higher WBC count, neutrophilia, lymphocytopenia and lower platelet levels. In particular, neutrophilia and lymphocytopenia act as markers of high death risks of patients with COVID-19. This is consistent with previous findings showing that severe patients with COVID-19 display higher neutrophil count and lower lymphocyte count during the period of diseases [13, 15] and that neutrophilia and lymphocytopenia are significantly associated with higher risks of the development of ARDS [6]. It has been showed that phagocytosis, release of granular contents, and secretion of cytokines are important effector functions of stimulated neutrophils, suggesting a protective immunity against the virus [16]. However, excessive elevated neutrophils can lead to severe pneumonia and death [16], which have been found in patients with SARS [17, 18] and MERS [19]. As for lymphocytes, it has been reported that the functional exhaustion of cytotoxic lymphocytes is associated with SRAS-CoV-2 infection, suggesting that SARS-CoV-2 infection may break down the antiviral immunity at an early stage [20]. Lymphocytopenia was also found in the MERS cases. Specifically, MERS-CoV can directly infect human primary T lymphocytes and induce T-cell apoptosis through extrinsic and intrinsic apoptosis pathways [21]. Therefore, it is possible that neutrophilia and lymphocytopenia are related, at least in part, to the development of critical illness, with a high mortality rate in the confirmed COVID-19 patients. Future studies to identify the mechanisms underlying these abnormal immune features will enhance our understandings of this disease.

It is well known that subsets of lymphocytes play important roles in the maintenance of immune system function. Here, our results indicated that the numbers of CD3^+^ T cells, CD4^+^ T cells, CD8^+^ T cells, B cells (CD19^+^), and natural killer (NK) cells (CD16^+^CD56) were significantly decreased in non-survivors at admission, compared with survivors. Meanwhile, increased serum concentrations of IgG, IgA, and IgE and decreased serum levels of C3 and C4 were found in non-survivors at admission. Taken together, these findings suggested that SARS-CoV-2 infection may impair lymphocytes, especially T lymphocytes, and the humoral immune system on the onset of the disease. The reduced abundance of CD4^+^ T cells, B cells, and NK cell count is likely a result of the rapidly propagating virus [22]. To mount an antiviral response, the innate immune system recognizes molecular structures that are produced by the invasion of the virus. Helper T (Th) cells orchestrate the overall adaptive response, while cytotoxic T cells are essential for killing of viral infected cells. Humoral immune response plays a protective role by limiting infection at later phase and prevents reinfection in the future. Therefore, SARS-CoV-2 infection can activate both cellular immune response and humoral immune responses in humans [23]. The uncontrolled inflammatory innate responses and impaired adaptive immune responses may lead to harmful tissue damage, both locally and systemically [24]. Patients who survived SARS-CoV and MERS-CoV infections usually had better immune responses than non-survivors [25].

It has been reported that CD4^+^ T cells and CD8^+^ T cells play a key antiviral roles by balancing the combat against pathogens and the risk of developing autoimmunity or overwhelming inflammation [26]. Depletion of CD4^+^ T cells, but not CD8^+^ T cells, leads to an enhanced immune-mediated interstitial pneumonitis and delayed clearance of SARS-CoV from the lungs in a SARS-CoV-infected mouse model, demonstrating the vital role of CD4^+^ T cells in preventing SARS-CoV infection [27]. A previous report has shown that higher number of CD4^+^ T cell may protect patients from developing ARDS in SARS-CoV infection [28]. In our study, we showed that the number of CD4^+^ T cells positively correlated with the numbers of lymphocytes and the level of oximetry saturation and negatively correlated with age and the numbers of neutrophils in non-survivors. Furthermore, low CD4^+^ T cell counts was a risk factor of death in patients with confirmed COVID-19. Together, our results provided an evidence showing that decreased number of CD4^+^ T cells is an immunity-related risk factor predicting death rate of patients with COVID-19. Similar to these findings, autopsy of patients with COVID-19 showed that the counts of peripheral CD4 and CD8 T cells were substantially reduced, which accounts for, in part, the severe immune injury in this patient [29]. High levels of pro-inflammatory cytokines released form CD4^+^ T cells have been observed in SARS-CoV and MERS-CoV infections as well, suggesting that the cytokine storm may contribute to disease severity [19]. Additionally, recruitment of immune cell populations in the patient’s blood was observed before the resolution of symptoms [30].

Although measurements of IL-6 levels have been performed in only some patients, our results indicated that non-survivors also had higher IL-6 levels as compared to survivors, raising the possibility that abnormal IL-6 levels may also contribute to in-hospital death [14]. IL-6 is a pro-inflammatory regulator of T cells. It can promote differentiation of naïve T cells towards Th17 cell lineage, inhibit the induction of regulatory T-cells (Tregs) [31] Additionally, Th17 cells can induce an inflammatory response through the production of IL-17A and IL-17F [32], which act as key cytokines for neutrophils migration, recruitment and activation [33]. It has been reported that activated neutrophils can phagocytosis, release granular contents, and produce cytokines, therefore contributing to a protective immune response against the virus [16] . However, excessive elevated neutrophils can lead to cytokine release syndrome (CRS) and tissue damage, resulting in severe pneumonia and death [16], which have been found in patients with SARS [17, 18] and MERS [19]. CRS is a systemic inflammatory response that can be triggered by infection and other factors and is characterised by a sharp increase in the level of a large number of pro-inflammatory cytokines [34]. SARS-CoV-2 binds to alveolar epithelial cells, after which the virus activates the innate and adaptive immune systems, resulting in the release of a large number of cytokines, including IL-6 [35]. It has been observed that there is a strong involvement of IL-6 in inducing CRS which corresponds to a higher rate of morbidity associated with SARS-CoV and MERS-CoV infections [36]. The signaling of IL-6 plays an important role in inducing this cytokine storm (both by its classical and trans signaling pathway) in severe COVID patients. Consequently, to control the effect of IL-6 and the resulting abnormal immunity, immunotherapies such as tocilizumab [37], vaccine against SARS-COV-2 [38, 39] and targeting toll-like receptor 5 (TLR5) [40] were developed.

The importance of complement in SARS-CoV pathogenesis is controversial. The complement system is an essential component of the innate immune system, and complement plays an important role in the host antiviral response [41]. C3 is required for protection from the pandemic 2009 H1N1 and the highly pathogenic avian influenza (HPAI) H5N1 influenza virus infections by aiding in viral clearance and regulating lung inflammation [42]. Activation of the complement system also results in an immune reaction capable of destroying pathogens and their products. C3 was an important host mediator of SARS-CoV-induced disease and it regulates a systemic proinflammatory response to SARS-CoV infection [43]. Here, our data showed that non-survivors displayed markedly decreased serum levels of C3 and C4 at admission when compared with survivors. Additionally, our results suggested that decreased serum level of C3 is the immunity-related risk factor predicting mortality of patients with COVID-19.

Our study has some limitations. First, we mainly evaluated the number change of cellular immunity- and humoral immunity-related cell subsets, whereas the function of these cells were no elucidated. Second, due to the retrospective study design, not all laboratory tests, such as IL-6, Interferon-γ, IL-10 and other cytokines, were performed in all patients, and the changes in data after treatment were incomplete.

## Conclusions

Abnormal cellular immunity and humoral immunity were key features in non-survivors with COVID-19. Additionally, we determined that neutrophilia, lymphocytopenia, low number of CD4^+^ T cells, and decreased level of C3 were the immunity-related risk factors that can predict mortality of patients with COVID-19. These new features and markers will shed light on developing new strategies for evaluating the prognosis of patients with COVID-19.

## Data Availability

The datasets generated and/or analysed during the current study are not publicly available but are available from the corresponding author on reasonable request.

## Abbreviations

COVID-19: Coronavirus disease 2019
WBC: White blood cell
CRP: C-reactive protein
PCT: Procalcitonin
Ig: Immunoglobulins
SARS-CoV-2: Severe acute respiratory syndrome coronavirus 2
SARS: Severe acute respiratory syndrome
MERS: Middle East respiratory syndrome
ARDS: Acute respiratory distress syndrome
CT: Computed tomograph
RT-PCR: Reverse transcriptase-polymerase chain reaction
CDC: Center for disease control and prevention
C: Complement proteins
IQR: Interquartile range
ROC: Receiver operating characteristic
CI: Confidence interval
NK: Natural killer
Th: Helper T
HPAI: Highly pathogenic avian influenza
IL: Interleukin
